# A Study of Redox Properties of Ceria and Fe-Ceria Solid Materials Through Small Molecules Catalytic Oxidation

**DOI:** 10.3390/ma18040806

**Published:** 2025-02-12

**Authors:** Riccardo Balzarotti, Andrea Basso Peressut, Gabriella Garbarino, Elena Spennati, Juan Felipe Basbus, Maria Paola Carpanese, Saverio Latorrata, Cinzia Cristiani, Elisabetta Finocchio

**Affiliations:** 1 Department of Innovative Technologies, University of Applied Sciences and Arts of Southern Switzerland, Via la Santa 1, 6962 Lugano, Switzerland; riccardo.balzarotti@supsi.ch; 2 Department of Chemistry, Materials and Chemical Engineering “G. Natta”, Politecnico di Milano, Piazza Leonardo da Vinci 32, 20133 Milan, Italy; andreastefano.basso@polimi.it (A.B.P.); saverio.latorrata@polimi.it (S.L.); 3 Department of Civil, Chemical and Environmental Engineering (DICCA), University of Genova (UniGe), Via All’opera Pia 15, 16145 Genoa, Italy; gabriella.garbarino@unige.it (G.G.); elena.spennati@edu.unige.it (E.S.); juanfelipe.basbus@edu.unige.it (J.F.B.); maria.paola.carpanese@unige.it (M.P.C.)

**Keywords:** ceria catalyst, impregnation, FT-IR spectroscopy, X-ray diffraction, Rietveld refinement, redox properties, environmental catalysis, temperature programmed surface reaction

## Abstract

This work presents a study of the redox properties of CeO_2_ particles with (FeCeHS) and without (CeHS) Fe_2_O_3_ impregnation, as possible innovative catalysts for oxidation and combustion reactions as well as CO_2_ activation. The topic, therefore, is part of a broader analysis of environmental catalysis, which aims to reduce the emissions of polluting substances and improve the exploitation of energy resources, with consequent progress in the eco-friendly field. Different laboratory techniques (Scanning Electron Microscopy (SEM), X-ray diffraction (XRD), Ultraviolet–Visible (UV-Vis), and Fourier Transform–Infrared (FT-IR) spectroscopies) point out that iron oxide is deposited on the surface of ceria, which maintains its lattice structure, although the particle morphology is slightly changed. Methanol and ethanol adsorption and conversion were evaluated on these catalysts by Temperature Programmed Surface Reaction (TPSR) and by in situ FT-IR spectroscopy of the probe redox properties, evidencing the formation of surface oxidized intermediates and combustion products. The FeCeHS catalyst demonstrates, in our reaction conditions, a good combustion activity in total oxidation of oxygenated molecules, hindering the formation of formaldehyde from methanol and reducing the quantity of CO produced by the partial oxidation reaction. A cooperative effect is suggested by the mixture of these two metals in the oxidation process.

## 1. Introduction

Catalysts based on pure and modified CeO_2_ have been widely applied in several fields, due to their high oxidation activity, excellent oxygen-storage capacity (OSC) properties, and stable redox behavior involving surface and sub-surface regions [1,2]. In addition to the well-known applications in three-way catalysts and in diesel engines [2], some recent exploitations of pure ceria and/or modified ceria range from oxidation catalysts for volatile organic compound (VOC) combustion and abatement [3,4] and methane oxidation [5], to CO oxidation [6], catalytic steam reforming [7], and CO_2_ catalytic activation [8]. Applications of ceria-based materials are also reported in solid oxide cells (SOCs) [9,10,11] and water thermocatalytic splitting [2], as well as in water electrolysis and hydrogen electro-oxidation [12].

The possibility of Ce reduction from 4+ to 3+, depending on the redox conditions of the environment, is the base of its high activity and flexibility in a wide range of applications. Moreover, in catalyst and electro-catalyst formulations, ceria can be doped with other cations to achieve a synergistic effect and further improve the redox behavior [13]. In this respect, iron oxides present interesting properties, such as high thermal resistance, low environmental impact, and availability in non-critical quantities in the earth’s crust and from industrial wastes [14]. The presence of small Fe cations (Fe^3+^/Fe^2+^) in mixed/doped ceria-based materials has been reported to increase the amount of Ce ions in oxidation state +3, thus favoring oxygen mobility at the catalyst surface.

Therefore, a number of Fe-modified ceria materials have recently received attention, mainly focused on several oxidation processes, involving methane [15,16], CO [17,18,19,20,21], soot [22,23], or other molecules [24,25,26,27,28,29,30], as well as a focus on the reduction of CO_2_ [31,32,33] or NO_x_ [34,35]. Fe-doped ceria catalysts have also been investigated for the degradation of organic pollutants [36,37,38] or to facilitate water splitting [39]. Perez-Alonso et al. [24] have verified that Fe-Ce mixed oxides, obtained by co-precipitation, demonstrated high activity and stability at high temperature when employed in the decomposition of N_2_O. Indeed, they have seen that the conversion rate of the oxide containing 50 at. % of cerium increases by roughly three times compared to that of pure iron oxide and by about six times with respect to that of pure cerium oxide. This behavior has been ascribed to the formation of a more stable solid solution, which has eventually led to a better reducibility of the Fe sites, thus enhancing the catalyst performance. Similar findings have been obtained by Li et al. [15], who prepared CeO_2_-Fe_2_O_3_ oxides by co-precipitation and tested them for the direct gas–solid conversion of methane to synthesis gas. These oxides exhibited a high lattice oxygen content, which turned into a high reduction peak, attributed to the consumption of H_2_ by bulk CeO_2_, and consequently into an excellent catalytic activity. CeO_2_-Fe_2_O_3_ oxides have also displayed a higher selectivity towards CO and H_2_ than CeO_2_-ZrO_2_ and ZrO_2_-Fe_2_O_3_ oxides, also tested in the work by Li et al. [15]. A similar catalyst, containing 60% (*w*/*w*) Fe_2_O_3_ and 40% (*w*/*w*) CeO_2_, has been prepared by Tang et al. [16] and demonstrated optimal Fe dispersion and surface area, leading to a high catalytic activity towards the decomposition of methane. Laguna et al. [18], instead, have exploited the microemulsion method to synthesize a series of stable Ce-Fe mixed oxides. They verified the presence of a slight segregation of α-Fe_2_O_3_ at 50% (*w*/*w*) iron content, while the mixed oxide containing 10% (*w*/*w*) Fe exhibited the highest concentration of oxygen vacancies and efficiency towards the oxidation of CO. Conversely, the effect of different production methods has been assessed by Wang et al. [26], who prepared Fe-doped CeO_2_ mixed oxide nanosheets via hydrothermal, cold co-precipitation, or solvothermal synthesis. The catalysts containing 5% (*w*/*w*) iron demonstrated the best properties for the combustion of 1,2-dichloroethane. However, those prepared via the hydrothermal method displayed the greatest catalytic activity below 175 °C, while between 175 °C and 350 °C, the best performance was given by the Fe-CeO_2_ catalyst synthesized by the solvothermal method. The latter provided the largest concentration of oxygen vacancies and surface-active oxygen but also showed a lower selectivity of polychlorohydrocarbon byproducts, whose formation was inhibited by increasing the Fe content up to 15 at. %. The role of oxygen vacancies in Fe-doped CeO_2_ oxides, employed for the oxidation of soot, has been studied by Li et al. [22]. They prepared the catalysts by means of co-precipitation or solution combustion synthesis, varying the iron content between 0 and 30% (*w*/*w*). Co-precipitation allowed an optimal activity to be achieved at 10 at. % Fe content, while the solution combustion method enabled a comparable performance to be obtained at 5% (*w*/*w*) Fe content. Furthermore, Li et al. [22] have found that, regardless of the fabrication method, the Ce^3+^ content, closely related to that of the oxygen vacancies, was the most important factor in determining the catalytic activity.

Ceria-based catalyst formulations have also been evaluated for their application in structured reactors. In fact, the catalytic systems most widely used in environmental applications are monoliths or foams, because they offer great advantages such as a lower pressure drop when high flow rates are required. Among others, the destruction of volatile organic compounds (VOCs) from chemical plants, domestic sources, and restaurants; the selective oxidation of alkanes; and catalytic combustion are examples of these processes [40]. Generally, the structured catalysts are prepared via a coating process, which implies the coverage of the surface of an inert support of complex geometry. The coating procedures of transition metal based-catalysts, supported on low surface area CeO_2_ powder (CeLS, 3 m^2^ g^−1^), have already been studied by some of this paper’s authors [41]. However, due to the complex geometry of the coated system, it is hard to deeply characterize the surface chemistry and redox activity of the catalytic powders, and it is common practice to perform a deep characterization of the powders before coating them on the geometrical support. In this respect, this paper intends to assess the structure, the redox properties, and the catalytic behavior of high surface Ceria and Ceria-supported iron oxide catalysts, to be used in the form of powders, in view of their possible application for deposition onto open-cell foam structured reactors.

The CeO_2_ and Fe/CeO_2_ catalytic powders studied in this work were produced according to the combined precipitation–dry–impregnation process developed elsewhere [42]. This synthesis route implies a double step process, where the support is first obtained via a precipitation reaction [42] and then is impregnated via the incipient wetness technique with a precursor of the active phase [43,44]. To the best of the authors’ knowledge, only a limited number of studies have been devoted to Fe/CeO_2_ impregnated systems [21,45]. The fully spectroscopic characterization through adsorption and activation of oxygenated probe molecules, coupled with the results from Temperature Programmed Surface Reaction studies, can give new useful insight into the surface activity and oxidation capability of these materials for possible applications in VOC oxidation processes.

## 2. Materials and Methods

As reported above, the Fe/CeO_2_ catalyst was produced according to the combined precipitation–dry–impregnation process already developed elsewhere [42,43].

Briefly, the CeO_2_ support (CeHS) was obtained by dropping a water solution of 2.6 M (NH_4_)_2_CO_3_ (Sigma-Aldrich, St. Louis and Burlington, MA) into a water solution of 1 M Ce(NO_3_)_3_⋅6H_2_O (Sigma-Aldrich) under magnetic stirring. The slurry was reacted at 333 K for 3 h; then, it was filtered under pressure, and the cake was washed and filtered 3 times until neutral pH. The resulting solid was dried at 393 K overnight and finally calcined at 673 K for 3 h (heating/cooling rate of 2 K min^−1^) [46,47].

The Fe-based catalyst (FeCeHS) was obtained via the incipient wetness technique, which implies the dropwise addition to the support of a solution of a soluble precursor of the active phase, i.e., Fe(NO)_3_⋅9H_2_O (98% pure, Sigma-Aldrich, St. Louis and Burlington, MA), dissolved in a water amount corresponding to the total pore volume of the support. The concentration of Fe in the solution and the number of impregnation steps were set to reach a nominal Fe content of 7% (*w*/*w*) of the total. After that, the solid was dried overnight at 393 K and calcined at 673 K for 10 h (heating/cooling at 2 K min^−1^).

The final Fe content of 7% (*w*/*w*) was confirmed with an estimated accuracy higher than 99% by chemical analysis via Inductively Coupled Plasma Optical Emission Spectroscopy (ICP/OES), (PerkinElmer OPTIMA 7000 DV spectrometer, Shelton, CT, USA).

Particle dimensions (Dps) were determined by means of laser granulometry (CILAS 1180 equipment, Orléans, Aubagne and Le Barp, France) [48,49].

The surface area (SA), pore volume (Vp), and pore radius (rp) were determined by N_2_ adsorption at 77.35 K (Micromeritics Tristar 3000 instrument, Norcross, GA, USA); before the analysis, samples were degassed overnight at 333 K (heating rate of 1 °C min^−1^ from 298 K to 333 K).

The crystallographic properties of the CeHS and FeCeHS powders were studied by X-ray diffraction (XRD) at room temperature (RT) in air. The PANalytical AERIS equipment, (Lissone MB, Italy) was operated at 30 kV and 10 mA, with Bragg–Brentano (reflection) geometry, Cu K_α_ radiation, angular range of 20–120°2θ, 0.011°2θ step size, Ni filter, and PIXcel^1D^ detector. The profiles of the XRD patterns were refined by the Rietveld method using the FullProf Suite software (Version 6 May 2024) [50]. A 6th degree polynomial, Thompson–Cox–Hastings pseudo-Voigt convoluted with axial divergence asymmetry function, and isotropic Debye–Waller factors were used for the background fit, peak profiles, and atomic thermal displacement, respectively. The Fm-3m (N° 225) space group was used as the seed for the CeO_2_ support, and the Wyckoff positions (WPs) for Ce and O were assigned as 4a and 8c, respectively. In the case of Fe_2_O_3_, the space group was considered as R-3c (N° 167), while Fe and O were ascribed to 12c and 18e, respectively. In this sense, the weight percentage of Fe_2_O_3_ was calculated and compared. The crystallite size (CS) and microstrain (MS) of the powders were estimated by using micrometric CeO_2_ powders as the XRD standard, due to their larger grain size (i.e., above 5 µm) and their peaks with narrower full width at half maximum (FWHM) with respect to the nanometric samples, while the 2θ positions are identical.

Fourier Transform–Infrared (FT-IR) spectroscopy studies of organic molecules were performed on pure powder disks activated in air and in vacuum at 673 K before adsorption experiments. Methanol and ethanol adsorption was performed at RT and, after a short outgassing step, upon increasing the temperature in static conditions. FT-IR spectra of the catalyst surface and gas phase were recorded at each temperature step. In all cases, a Nexus ThermoNicolet instrument equipped with DTGS detector (Waltham, MA, USA) was used (OMNIC 3.2 software, 100 scans). Skeletal IR studies have been performed on the same instrument on the catalyst powders diluted in KBr.

Ultraviolet/Visible/Near Infrared (UV-Vis, NIR) spectroscopy was carried out with a Jasco V570 spectrometer, (Easton, MD 21601, USA) equipped with a diffuse reflectance (integrating sphere) for powder analysis.

The microstructure and elemental composition of the CeHS and FeCeHS powders were studied by Scanning Electron Microscopy (SEM) using a Phenom ProX apparatus, coupled with an Energy Dispersive Spectroscopy (EDS) detector (Waltham, MA, USA). SEM images as well as EDS spectra were collected by using a CeB_6_ source operated at 15 kV in ultra-high vacuum (UHV). The EDS detector presents an accuracy better than 0.2 wt.%, energy resolution above 0.13 keV, and atomic-number, absorption, and fluorescence (ZAF) correction. Both point analysis and elemental mapping were performed.

Temperature Programmed Surface Reaction (TPSR) experiments were conducted at atmospheric pressure in a tubular flow reactor using 0.043 g of catalyst diluted in quartz sand. The feed composition in the different tests was set as follows: 1% (*v*/*v*) ethanol (99.8% assay, from Sigma Aldrich, St. Louis and Burlington, MA, USA) and 6% (*v*/*v*) oxygen, or 1.7% (*v*/*v*) methanol (98%, from Sigma Aldrich, St. Louis and Burlington, MA) and 2.9% (*v*/*v*) oxygen, diluted in nitrogen, which was used as the gas carrier. The total flow rate was set at 170 mL min^−1^. During a typical activity test, the catalytic bed was preheated to 373 K in pure nitrogen (flow rate of 170 NmL min^−1^). Subsequently, a nitrogen/air mixture was fed to the system under the same reaction conditions before switching to the required reaction feed. The outlet gases were analyzed by FT-IR, using a Thermo-Nicolet 380, (Waltham, MA, USA) (DTGS detector, 4 scans). The wavenumbers of the diagnostic bands of the analyzed reactants and products are reported in previous works [51]. The reactor temperature was increased stepwise by 5 K min^−1^ from 373 K up to 773 K. For the sake of completeness, blank tests were carried out by using only quartz sand. They showed the formation of acetaldehyde and ethylene in traces at 773 K when ethanol was fed, while no reaction products were detected upon feeding methanol.

## 3. Results and Discussion

### 3.1. Material Characterization

#### 3.1.1. Textural Properties and Phase Composition

The textural properties of both the CeHS support and FeCeHS catalysts are summarized in Table 1. A trimodal distribution of the particle size was found for both powder samples; however, FeCeHS is characterized by slightly lower particle dimensions, suggesting the presence of a somewhat disaggregating effect during the wetness impregnation.

The CeHS support was characterized by a surface area of 90 m^2^ g^−1^, which is in line with the range typically reported in the literature for mesoporous Ce-based samples when prepared via an inorganic synthetic route without using pores formers [21]; therefore, the textural properties make this material suitable for its use as a support for catalysts.

Once impregnated, a marked decrease of the SA down to 53 m^2^ g^−1^ was observed, accompanied by an increase in the mean pore radius from 4 to 9 nm (Table 1).

For materials of similar composition and prepared via a similar synthetic route, the incorporation of iron was reported as responsible for the decrease in both the pore volume and average pore diameter [21], but this behavior has not been observed for the FeCeHS sample. Therefore, the combined effect of both double calcination at 673 K, required for the preparation, and pore clogging due to the presence of Fe in the pores upon impregnation can be assumed to explain the textural modification observed in FeCeHS.

**Figure 1:**
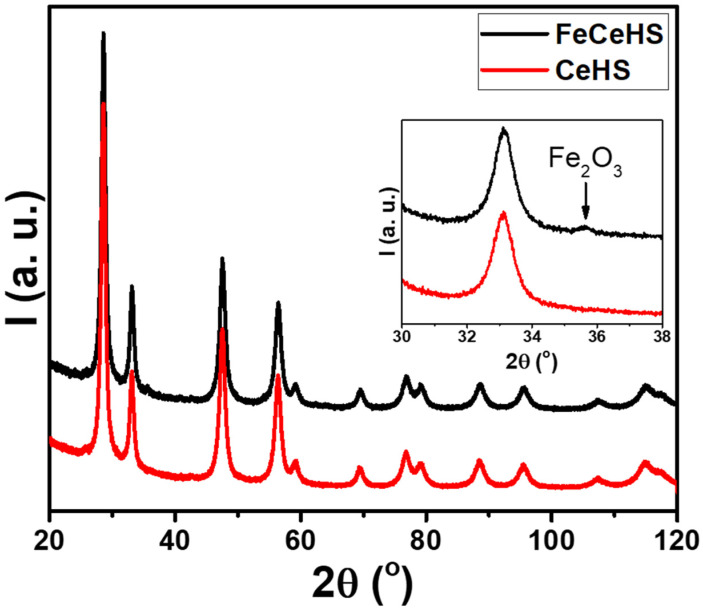
X-Ray diffraction patterns of CeHS and FeCeHS. The intensity (I) is expressed in arbitrary units (a. u.); arrow: Fe_2_O_3_ reflection.

The X-ray diffraction (XRD) patterns of CeHS and FeCeHS are compared in Figure 1. The CeHS pattern evidenced the typical reflection of the CeO_2_ fluorite structure (JCPDF 00-004-0593); CeO_2_ is still the main phase detected in the FeCeHS pattern, accompanied by the presence of an iron-containing phase, possibly Fe_2_O_3_ (JCPDF 00-033-0664). Due to the overlapping of the reflections of the two phases, the presence of iron oxide is only indicated by the broad and low intensity peak at about 36 2θ [52].

For samples of similar composition and synthetic route, the formation of finely dispersed Fe oxides has been reported [21,25]. Indeed Lykaki et al., by performing XRD analysis of impregnated Fe_2_O_3_/CeO_2_ samples, reported the presence of highly dispersed hematite and demonstrated a homogeneous surface distribution of this phase by TEM and SEM-EDX [21]. Similarly, Reddy et al., on the basis of the Fe 2p photoemission spectra (XPS) of a 5% Fe/CeO_2_ solid solution, reported the formation of finely dispersed iron oxide species, which were associated with an Fe_2_O_3_ monolayer [25]. Accordingly, the presence of highly dispersed Fe oxide can also be hypothesized for the FeCeHS samples.

Both the CeHS and FeCeHS patterns were analyzed by Rietveld refinement. The results are summarized in Table 2, while the fitting parameters and the corresponding figure are reported in the Supplementary Materials (Table S1 and Figure S1, respectively).

The support and the catalyst are characterized by very close lattice parameters, 5.413(1) Å and 5.409(1) Å for CeHs and FeCeHS, respectively; hence, according to the literature, no incorporation of Fe ions in the CeO_2_ lattice has occurred [45]. These lattice parameters present an excellent agreement with those reported by Prieur et al. [53] and Gu et al. [54], according to quite similar crystallite sizes. The hematite/ceria interphase, although very limited in our case, would promote a strain and lattice parameter shrinkage related to their crystal structures. Moreover, these effects should be sensitive in nanometric crystallites (where 10 nm would correspond to 20 ceria unit cells), and Prieur et al. [53] discussed that the lattice parameter variation is also related to several other factors, namely, the formation of oxygen vacancies and surface stress resulting from the difference in coordination.

In Table 2, the very slight decrease in the CeO_2_ cell parameters in the FeCeHS sample could also suggest the formation of a solid solution. Solid solutions are, indeed, reported to form when bulk materials are prepared, for instance, via sol-gel or coprecipitation, when a calcination treatment is performed at least at 873 K [55], while for synthesis from preformed powders, a calcination at 1773 K is needed [56]. As a matter of fact, FeCeHS has been prepared according to the incipient wetness impregnation, implying the allocation of Fe ions at the surface of the support, and a calcination temperature of 673 K, which, in principle, is not enough to induce the formation of a bulk solid solution. Therefore, on the basis of the considerations above, a real structural modification of this sample is not envisaged.

This picture is also supported by the microstrain values of CeO_2_ in CeHS (0.562%) and in FeCeHS (0.582%); indeed, the higher microstrain value of CeO_2_ in the presence of Fe_2_O_3_ can be related to the mismatch between the lattice planes of the oxides at the surface.

Very similar crystal size values were obtained for CeO_2_ in the pristine support and in the catalyst, namely, 9.1 nm for CeHs and 9.7 nm for FeCeHS, which suggest that the reported effect of Fe ions on the decrease in the CeO_2_ nanodomains was apparently not manifested in our FeCeHS sample [21]. However, in view of the double calcination at 673 K, to which the support was subjected following the impregnation with Fe, an increase in the size of the ceria crystallites would have been expected.

It is reported in the literature that, for microcrystalline fluorite-type CeO_2_ materials, the sintering process occurs at the grain boundaries, and it is accompanied by pore elimination, thus resulting in a densification process. The presence of extra ions inhibits boundary diffusion, thus slowing the sintering process [21,57].

The elimination of the smaller pores upon the double calcination at 673 K is suggested by the larger mean pore radius of FeCeHS compared with the pristine support, namely, 9 nm compared with 4 nm (Table 1). Therefore, the apparent lack of sintering of the cerium phase in FeCeHS could be in line with a possible effect of the Fe ions, which slow down the sintering process while interacting with the surface. As a matter of fact, the calcination temperature of 673 K applied to FeCeHS was too low, compared to 773 K reported in the literature [21], to bypass the activation energy for the Ce ion diffusion at the grain boundaries. Thus, the expected decrease in the CeO_2_ crystal size was not manifested. Accordingly, the marked decrease in the surface area observed in FeCeHS can be associated with the sintering effect during thermal treatment and the associated porosity decrease [57].

Considering the Fe-containing phase, in view of the impregnation method, lower crystal size and higher microstrain values are expected; however, the overlapping phases and the low intensity of the reflections prevented the determination of the microstructural parameters. Nevertheless, the calculated amount of Fe in the sample, i.e., 7%, is in good agreement with the actual Fe content.

The skeletal FT-IR spectra of the two materials are reported in Figure S2. The main absorption in the low frequency region falls at about 450 cm^−1^ (CeHS sample) and is assigned to vibrational modes typical of ceria’s structure. A small shift in the wavenumber can occur due to the different shapes and sizes of the particles [58] and to the presence of other metal oxides. In the FeCeHS sample spectrum, absorptions due to Fe-O vibrational modes are barely detectable as high frequency shoulders of the main absorption band of the spectrum (470 cm^−1^, tailing to 720 cm^−1^). The sharp band at 1380 cm^−1^ can be assigned to some residual nitrate from the preparation procedure.

**Figure 2:**
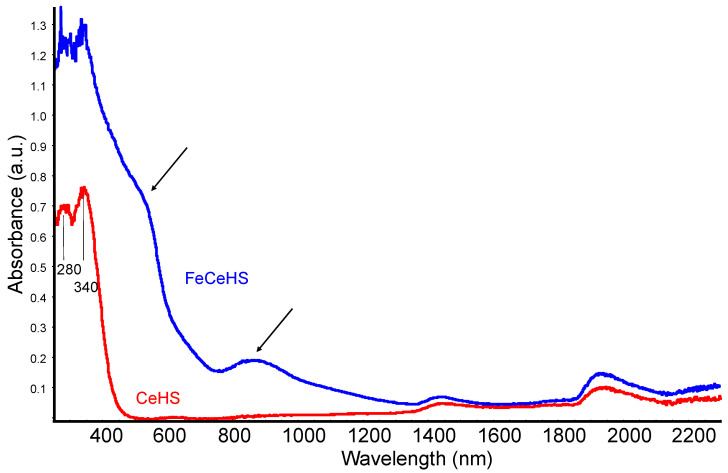
UV-Vis-NIR diffuse reflectance spectra. Arrows: shoulder 400–600 nm, and 800–900 nm.

The UV-Vis diffuse reflectance and near infrared (NIR) spectra are shown in Figure 2. The spectrum of the CeHS sample (Figure 2) presents the typical strong absorption below 400 nm, divided into two broad components and corresponding to the charge transfer O^2−^ (2p)--> -Ce^4+^ (4f). The sharp decrease in the absorption wavelength between 400 and 450 nm corresponds to the onset of the fundamental absorption edge [59]. The main feature below 400 nm, split into the two components, is still detectable in the FeCeHS sample spectrum. Moreover, the addition of FeO_x_ extended the sample absorption in the visible region between 450 and 800 cm^−1^, and the absorption edge shifts to higher wavelengths, likely due to the charge-transfer transition between the d-electrons of Fe ions and CeO_2_ conduction or valence bands [52,60].

The broadness of the absorption, appearing as a shoulder between 400 and 600 nm, in the FeCeHS spectrum, makes it difficult to identify in detail the overlapping components of this adsorption (evidenced by arrows in Figure 2). For instance, according to Gálvez et al. [61] and Torrent et al. [62], the shoulder between 400 and 600 nm can be attributed to the superposition of the absorption bands of the Fe^3+^ ligand field transitions in hematite and goethite at 405, 425, 435, 445, 488, and 538 nm. In particular, the bands at 405 and 445 nm were assigned to hematite electron transitions, while the one at 538 nm was related to the Fe^3+^ → Fe^3+^ pair excitations in hematite. Moreover, the bands at 425, 435, and 488 nm are related to the Fe^3+^ → Fe^3+^ magnetic coupling between edge-sharing octahedra in goethite. Furthermore, according to the literature and considering the complexity reported above, it is not possible to distinguish between isolated or aggregated Fe^3+^ species [25].

A possible alternative explanation implies the copresence of hematite with Fe_3_O_4_ inverse spinel. Indeed, studies on iron-containing minerals reported that the transition bands of the tetrahedrally coordinated Fe(II) ions occur at wavelengths (689, 613, and 562 nm [63]) that are slightly higher than those of tetrahedrally coordinated Fe(III) (378, 458, 520, and 550 nm [63]). Accordingly, the broad band between 450 and 650 nm could also be consistent with Fe_3_O_4_ inverse spinel structure, where Fe^3+^ cations occupy 1/8 of the tetrahedral interstitial “A” sites in the cubic close-packed oxygen lattice, while a 50:50 mixture of Fe^3+^ and Fe^2+^ cations occupies one-half of the octahedral “B” sites [64]. Furthermore, the broad band observed at about 850–900 nm can be associated with the Fe^2+^/Fe^3+^ IVCT (intervalence cation charge transfer) band [65]. The presence of Fe^2+^/Fe^3+^ IVCT is also supported by the red-orange color of the sample resulting from this phenomenon, thus confirming the co-presence of Fe(II)/Fe(III) ions allocated in the sample. The presence of some Fe^2+^ species in Fe/CeO_2_ samples has already been reported on the basis of XPS investigation. It was suggested that they were formed by the interaction between CeO_2_ and Fe_2_O_3_ via an interfacial redox process [21].

The band gap for these two materials has been evaluated through the Tauc plot presented in the Supplementary Materials (Figure S3) [66,67]. Indeed, the value near 3.1 eV obtained for pure ceria is consistent with the literature data [68]. The addition of iron leads to a significant decrease in the band gap value to about 2.7 eV. These values are also consistent with the ones reported for doped ceria nanomaterials prepared by methods other than dry impregnation [69]. The lower band gap energy has been related to an increased number of oxygen vacancies associated with the transition metals’ presence [70]. Other broad and weak bands detected in the NIR region above 1400 nm are assigned to overtones and combination modes of OH groups.

**Figure 3:**
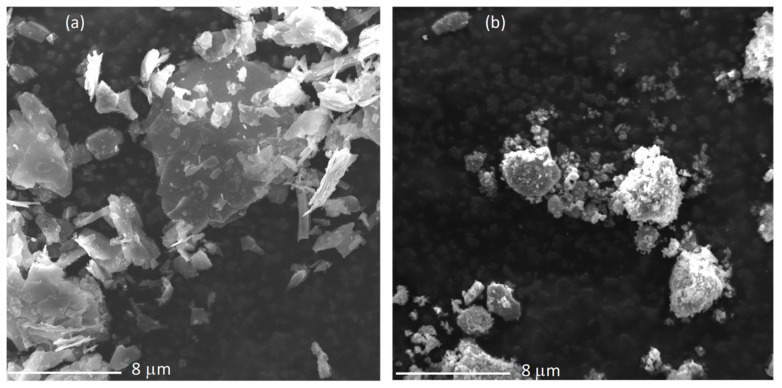
SEM images of (**a**) CeHS and (**b**) FeCeHS.

The SEM images of pristine ceria and Fe-impregnated samples are presented in Figure 3. Pure ceria apparently consists of lamellar particles (Figure 3a), while this configuration is partially lost by the Fe addition, after which the particles showed a spherical shape, with indented edges (Figure 3b). This morphological modification could be due to a possible variation in the surface charge, since the Fe(NO_3_)_3_ impregnating solution is slightly acid and the particle–particle interactions could be modified. From an inspection of the SEM images, particle dimensions of about 8–9 µm are present in CeHS, while those of 3–4 µm are found for FeCeHS, thus confirming the dispersion effect observed by laser granulometry (Table 1).

However, SEM analysis did not allow the determination of the crystallite shape, which can be of paramount importance in the assessment of the material properties. Indeed, it is reported in the literature that the particle shape can influence the textural properties in Fe_2_O_3_/CeO_2_, which ultimately can control the reactivity of the system, being the catalytic activity shape-dependent. Accordingly, for Fe_2_O_3_/CeO_2_ systems prepared in a similar way, on the basis of TEM analysis, the possible presence of nanocubes, nanopolyhedra, and nanorods has been reported, and the textural properties were correlated with the particle shape. By comparing the CeHS and FeCeHS textural properties with those reported in the literature, it can be hypothesized that our samples mainly consist of mixed nanocubes and nanopolyhedra [21].

The EDS analysis, intended here as a semiquantitative analysis (Table S2), is in good agreement with the actual Fe_2_O_3_ content. Traces of N and C are present, due to the presence of residual undecomposed nitrates and surface carbonates, as revealed by FT-IR spectroscopy [71].

#### 3.1.2. Surface Characterization

**Figure 4:**
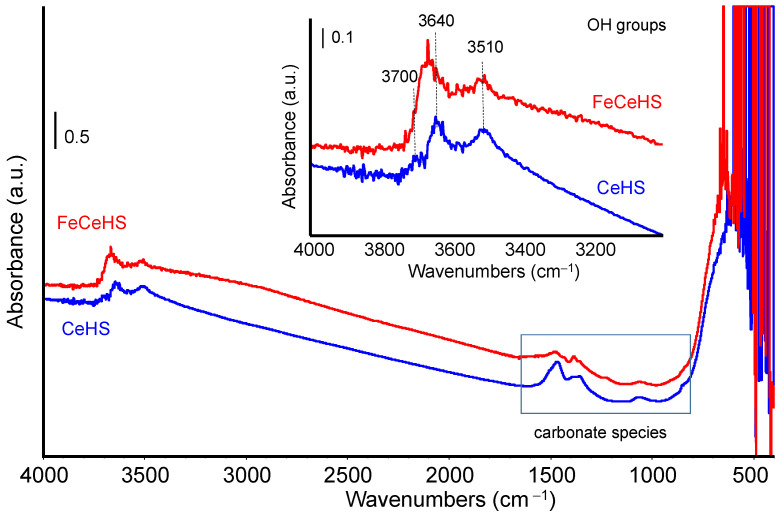
FT-IR spectra of pure powder disks outgassed at 673 K. Inset: enlargement of the OH stretching region.

Figure 4 shows the FT-IR spectra of the pure powder catalysts activated in vacuum in situ at 673 K. The spectrum of pure ceria exhibits two weak and broad bands centered at 3700 cm^−1^ and 3640 cm^−1^, assigned to hydroxyl groups that are linear and bridged on Ce^4+^ cations [72]. The broad component at around 3510 cm^−1^ may be due to residual adsorbed water or bicarbonate species, very likely to appear at the relatively low activation temperature applied in these experiments.

The presence of iron oxide leads to a broadening and shift to high frequencies of the terminal OH band, which is now centered at 3670 cm^−1^. This effect can be caused by the presence of exposed hydroxyl groups coordinated on Fe ions and, possibly, by the formation of new OH groups bridging both Fe/Ce ions. At the same time, the high frequency band detected in the spectrum of pure ceria seems to disappear. Indeed, the high frequency of this band corresponds to terminal OHs that can be located over the corner and/or edges of the particles. These OHs are in a lower coordination state, thus more prone to be affected by doping with oxides. Moreover, due to the complexity of this spectral feature band, we cannot rule out the presence of Ce^3+^ ions or Ce ions near oxygen vacancies. In the low frequency region of the spectrum, a series of peaks due to adsorbed carbonates and hydrogen carbonate species can be detected, formed by the reactive adsorption of CO_2_ from the atmosphere over basic surfaces such as ceria. In the FeCeHS sample spectrum, these bands are clearly reduced in intensity and complexity, suggesting that iron oxide modulates the surface acidic/basic properties of ceria, causing a decrease in basicity. The overall surface acidity of ceria-based materials is defined by Lewis sites, as also reported in [58].

### 3.2. Surface Reactivity: Alcohol Adsorption and Conversion

In the following set of experiments, the surface reactivity has been tested through the adsorption of alcohols, whose conversion has been analyzed in situ in the IR cell both in the gas phase and at the surface.

#### 3.2.1. Methanol Adsorption and Conversion

**Figure 5:**
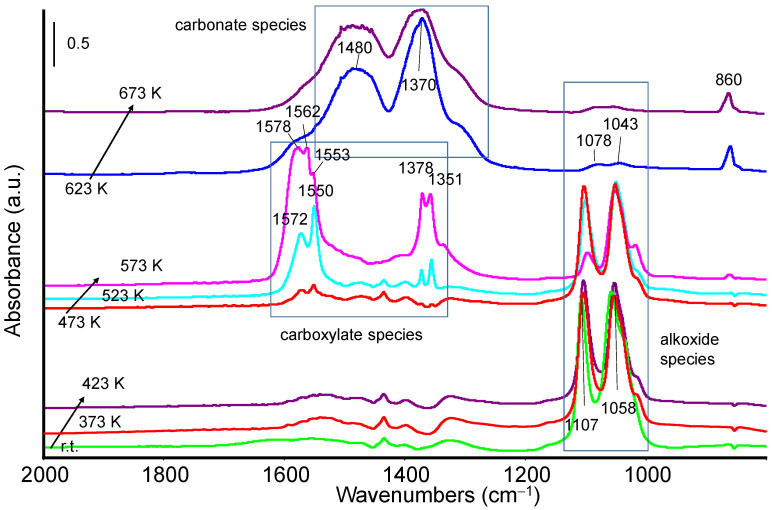
FT-IR subtraction spectra of surface species arising from methanol adsorption over the CeHS catalyst. The activated surface spectrum has been subtracted.

Following methanol adsorption at room temperature over the CeSH catalyst, several types of methoxides can form on the surface from the dissociative adsorption of the alcohol, depending on the nature of their bond with the surface (linear bridging or triple bridging) and on the oxidation state of the exposed Ce ions. In general, the CH_3_-O^−^ stretching vibrational mode, diagnostic of these interactions, can be detected in the spectral range of 1200–900 cm^−1^ [73]. In the spectra shown in Figure 5, the main bands associated with the formation of methoxides are centered at 1107 cm^−1^ and 1058 cm^−1^, assigned, respectively, to on-top and doubly bridging methoxy species coordinated over Ce^4+^ ions. The shoulder at 1115 cm^−1^ is due to triply bridging methoxy species. Correspondingly, the weak band at 1435 cm^−1^ is assigned to the CH deformation mode of the methyl group belonging to on-top coordinated methoxy species.

Following outgassing at increasing temperatures, the intensity of these peaks slowly decreases: first the on-top configuration, then the bridging species, which are more stable at the surface. At 473 K, formate species appear, characterized by bands at 1572 and 1550 cm^−1^ (ν_as_COO), 1378 (δCH), and 1351 cm^−1^ (ν_sym_COO). A further increase in temperature at 373 K leads to the detection of three stretching bands at 1578, 1562, and 1553 cm^−1^, suggesting the formation of at least three kinds of adsorbed formates. Early literature results assigned these bands to a mixture of formates adsorbed over reduced Ce^3+^ ions (1578 and 1562 cm^−1^), and formates adsorbed over Ce^4+^ ions (1550 cm^−1^), likely due to a spillover effect of these mobile species [73]. Thus, starting from 523 K, the oxidation of CH_3_O^−^ species to HCOO^−^ species occurs, in parallel to the Ce^4+^-Ce^3+^ reduction at the catalyst surface, confirming that Ce^4+^ ions are active sites in the oxidation process. In our reaction conditions, there is no evidence of formaldehyde species being formed at the surface. At 623–673 K, organic species almost completely disappeared, while broad and strong carbonate bands are detected in the spectra centered at 1480, 1370, and 860 cm^−1^. These results demonstrate the complete oxidation of the organic component and the consequent formation of carbonates as the final oxidation step by consuming the lattice oxygen of ceria. In these conditions, it is also possible to notice a continuous shift of wavenumbers for the bands associated with the residual methoxy species from 1107 and 1058 cm^−1^ (room temperature) to 1078 and 1043 cm^−1^ (673 K). Finally, the formation of carbonate species, i.e., the most oxidized species from organic molecules, parallels the detection of these two bands. The former band has been assigned to methoxy species bridging two Ce^3+^ ions (with or without nearby oxygen vacancies) thus indicating the partial reduction of the oxide surface [74].

The CH stretching vibrational modes of adsorbed methanol fall in the region of 3000–2700 cm^−1^ (Figure S4). The characteristic bands of methanol are found centered at 2908, 2885, and 2802–2798 cm^−1^ up to 473 K. They decrease in intensity as the temperature increases, and a new component grows at 2835 cm^−1^ above 523 K. Moreover, at 623 K, a peak centered at 2115 cm^−1^ is highlighted, due to CO coordinated over exposed surface metal ions. Finally, bands corresponding to the stretching of the OH group appear as negative features as a consequence of OH interactions with the organic substrate.

**Figure 6:**
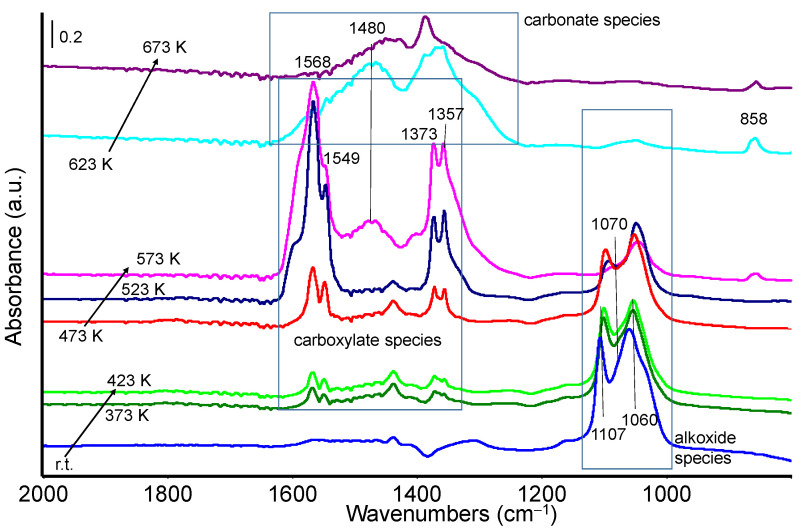
FT-IR subtraction spectra of surface species arising from methanol adsorption over FeCeHS. The activated surface spectrum has been subtracted.

The adsorption test with methanol was also carried out on the Fe-ceria catalyst, and the IR spectra are shown in Figure 6. Again, the formation of adsorbed methoxide is pointed out by the detection of bands in the region of 1200–1000 cm^−1^, whose intensity decreases at increasing temperature. Maxima are detected at 1107 and 1060 cm^−1^, consistent with the bands of methanol on pure ceria. Moreover, a shoulder growing around 1070 cm^−1^, typical of the Fe-containing sample, can be assigned to adsorbed methoxy species over Fe ions and is completely consistent with the results reported in the literature on the adsorption of methanol over α-Fe_2_O_3_ [75,76]. The shoulder at 1116 cm^−1^, reported for methoxy species on pure ceria, and due to triple bridging species, is not detectable anymore in this spectrum. Simultaneously, the OH stretching bands of surface hydroxyl groups disappear, as shown in the subtraction spectrum, as they are involved in H-bonding with undissociated methanol species.

These results are an indication of the exposure of Fe active sites at the surface, on the one hand, adding new adsorption sites, and on the other hand, limiting the availability of three Ce ions nearby to allow coordination of methoxy groups.

These findings might be indirect evidence of the high dispersion of iron oxide particles. Formate species, characterized by bands at 1568, 1549, 1373, and 1357 cm^−1^ are already detectable between 373 K and 423 K, i.e., lower temperatures than those observed with the pure ceria catalyst. The comparison of the spectra also shows that the asymmetric stretching band of formate species exhibits only two components, centered at frequencies that are slightly different from those of formates on pure ceria discussed previously. Thus, formates adsorbed over Ce^3+^ ions are detectable (band at 1560 cm^−1^, with a shoulder at higher frequency), while the band due to formate adsorbed over Ce^4+^ is reduced in relative intensity. This can be due to the presence of exposed iron oxide sites, which favor the formation of Ce^3+^ ions. The intensity of the peaks associated with formates increases with temperature, reaching its maximum at 573 K. At this temperature, on-top adsorbed methoxy species disappear almost completely and faster than on the ceria surface. Carbonate species are already forming (see the bands growing at 1480 and 858 cm^−1^) and become predominant in the spectrum at 623 K, as for the pure ceria catalyst. Apparently, there is a change in the nature of these species from 623 to 673 K, which leads to a shift of the IR bands towards lower frequencies.

Analyzing the residual adsorbed methoxy species, it seems that at high temperature, there is also a shift of the band position as reported in the previous paragraphs for methoxy on ceria. However, the component assigned to methoxy groups coordinated on iron ions (CO stretching band around 1070 cm^−1^) is still evident, suggesting the hypothesis that the Ce ions are the main active sites, while Fe ions enhance the oxidation activity of ceria.

Therefore, over FeCeHS catalysts, adsorbed methoxy species are quickly oxidized to carboxylates over Ce^4+^ active sites, and this step occurs at a lower temperature than on the pure ceria catalyst. On the other hand, the evolution of carboxylates to carbonate (total combustion) is similar for both the FeCeHS and CeHS materials. This result could explain the fact that partial oxidation products such as carbonyl species and CO are almost not detected after doping with iron, although both catalysts are effective oxidation catalysts.

Figure S5 shows the spectra of adsorbed methanol in the high frequency region. Up to 473 K, the characteristic CH stretching bands centered at 2908, 2884, and 2803 cm^−1^ can be recognized. By further increasing the temperature, there is clearly a decrease in their intensity, which drops to zero at 623 K. Again, the bands relating to the stretching of the hydroxyl groups appear as negative in the subtraction spectra, following interaction through H-bonds with alcohol molecules. It is worth noting that no peaks due to coordinated CO molecules are recorded in the range of 2200–2000 cm^−1^.

#### 3.2.2. Ethanol Adsorption and Conversion

**Figure 7:**
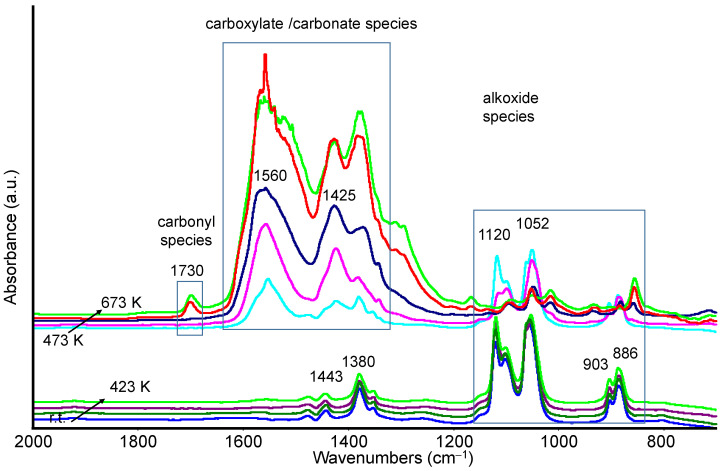
FT-IR subtraction spectra of surface species arising from ethanol adsorption over the CeHS catalyst. The activated surface spectrum has been subtracted. Different colors of the spectra correspond to the different (increased) temperatures within the range evidenced.

Reactive ethanol adsorption at room temperature over the CeHS surface gives rise to two groups of complex bands in the 1180–900 cm^−1^ region (Figure 7), due to the overlapping of CC and CO stretching modes of alkoxides groups. At 100 °C, maxima are centered at 1120 cm^−1^ (with a shoulder at 1100 cm^−1^) and at 1052 cm^−1^ (shoulder at 1060 cm^−1^) due to the asymmetric stretching vibration of the CCO system. The weaker bands at 903 and 886 cm^−1^ are due to the symmetric stretching vibration of CCO groups. The weak shoulder at 1148 cm^−1^ can be assigned to a CH_3_ deformation mode, having a partial CO stretching character. Bands due to CH deformation modes are also detected at 1380 and 1443 cm^−1^ [77,78]. A detailed assignation has been proposed by Podobinski et al. [79], specifically for ethoxy groups on ceria. Based on their paper, the bands at 903, 1060, and 1120 cm^−1^ characterize monodentate coordinated species, while the bands at 886 and 1055 cm^−1^ have been assigned to bidentate species, with both species coordinated over the oxidized ceria surface, i.e., Ce^4+^ ions. However, the formation of tri-coordinated ethoxy groups is not evident in our spectra. The intensity of all these peaks decreases with an uneven trend at increasing temperature. Between 523 K and 573 K, the decrease involves the components at higher frequencies, leaving bands at 1094, 1052, 1015, and 930 cm^−1^. At 623 K, the persistence of the band at 1052 cm^−1^ agrees with its assignation to residual bidentate ethoxy groups, which are resistant to outgassing at such high temperature and are coordinated over the ceria surface partially reduced by the oxidation of ethanol to carboxylate species [79].

Correspondingly, at 473 K, new bands appear in the 1600–1350 cm^−1^ region assigned to a mixture of strongly adsorbed carboxylate and carbonate species. In detail, the bands centered at 1560, 1425, and 1380 cm^−1^ are assigned to the symmetric and asymmetric stretching of the -COO^−^ group, overlapped with the deformation modes of the C-H bond of acetate species [80]. In addition, in this case, the complexity and broadness of these bands suggest the formation of several carboxylate complexes. The difference between the frequencies of the main maxima (i.e., 1560 and 1425 cm^−1^) is lower than the value reported for the “free” acetate ionic complex. Thus, it is reasonable to assume that the main acetate species adsorbed at this surface are bidentate/bridging complexes [81]. At 623 K, these bands are the main ones of the spectrum overlapped with new strong absorptions. New peaks are also highlighted in the 1000–850 cm^−1^ range, likely due to the COO deformation modes of adsorbed carbonates, i.e., the final oxidation step of organic species [82].

The high frequency region of the same spectra is reported in Figure S6. Up to 523 K, the characteristic bands of ethoxy groups are found centered at 2960, 2924, 2842, and 2700 cm^−1^, corresponding to CH stretching modes. The bands of OH groups appear as negative, at 3655 and 3685 cm^−1^, highlighting the consumption of these species during the conversion of ethanol. Moreover, at 573 K, a peak centered at 2120 cm^−1^ is detected, due to the formation of carbon monoxide (partial oxidation product) and its adsorption over both Ce and Fe ions exposed at the surface.

In addition to the main features discussed above, in the high temperature range (623–673 K), it was also possible to detect a peak at 1730 cm^−1^ assigned to the C=O bond of aldehyde. The analysis of the IR spectra recorded in the corresponding gas phase showed, in the same temperature range, the presence of aldehyde molecules, which indeed are reabsorbed at the surface in the static conditions of the experiments and cannot be further oxidized to carboxylates and/or carbonates. This is further confirmation that, at this stage of the reaction, the surface has already been at least partially reduced by the reaction of the organic molecules.

**Figure 8:**
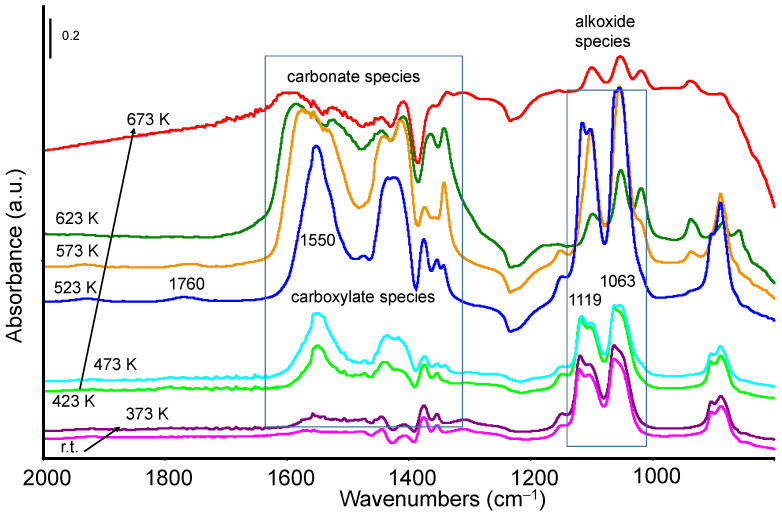
FT-IR subtraction spectra of surface species arising from ethanol adsorption over FeCeHS catalyst. The activated surface spectrum has been subtracted.

The adsorption of ethanol on the FeCeHS sample also leads to the formation of ethoxy species, characterized by bands in the 1200–1000 cm^−1^ region with maxima at 1119 and 1063 cm^−1^ and shoulders at 1104 and 1054 cm^−1^ (Figure 8). These features are almost the same as those detected in the spectra of pure ceria, but the relative intensities are changed. For instance, the band at 1063 cm^−1^ is predominant, suggesting an increased amount of monodentate species on ceria. Indeed, the presence of ethoxy groups coordinated on Fe ions cannot be ruled out. Starting already from 373 K, carboxylates, namely, acetates, are formed, characterized by bands in the 1550–1340 cm^−1^ region, which reach a maximum intensity at 523 K. At higher temperatures, adsorbed surface carbonates are detected, with bands at 1580–1570 cm^−1^ and 1410–1390 cm^−1^, whose intensity increases up to 350 °C before their decomposition. These findings point out a similar oxidation pathway of alcohol molecules over ceria and doped ceria. All the experiments have been carried out without oxygen in the gas phase; thus, for both catalysts, the oxidating agents are nucleophilic oxygen species (lattice oxygen), as we discussed and proposed in an earlier publication [83].

The addition of iron has significantly reduced the formation of carbonyl species in the oxidation pathway: a very weak C=O stretching mode is only detectable at 1760 cm^−1^ at 523 K. Moreover, the greater complexity of the spectra recorded for the FeCeHS sample can be explained considering that the breaking of the C-C bond in small organic molecules also occurs, depending on the reactivity of the surface. The peak at 1385 cm^−1^, growing between 573 and 623 K, can actually be assigned to the CH deformation mode of formate species [81].

In the high frequency region (3000–2700 cm^−1^) (Figure S7), as in the previous case, the characteristic bands of CH stretching modes of ethanol are detectable at 2962, 2924, 2862, and 2692 cm^−1^. The bands associated with the stretching of the OH group appear as negative features; however, their shape and position differ slightly in comparison to the same bands reported for the CeHS catalysts. This result confirms that exposed OH groups are both affected by iron doping and involved in the interaction with adsorbed molecules. Finally, between 523 K and 623 K a weak band is barely detectable at 2125 cm^−1^, due to the stretching mode of CO adsorbed on exposed surface ions and formed as a byproduct in the oxidation process. Its relative intensity, however, is significantly lower than in the case of pure ceria.

The spectra of gas phase species have been recorded in parallel to the spectra of the surfaces discussed above but are not reported here. In sum, a limited amount of ethylene arising from elimination reactions can be detected for both catalysts (peak centered at 950 cm^−1^ starting from 573 K [78]). Several other bands can be detected at 1305 cm^−1^ (methane), present only in the CeHS test; at 1734 cm^−1^ (acetaldehyde, clearly detectable in the CeHS test); and at 2350 cm^−1^ (CO_2_), present in both tests.

**Scheme 1:**
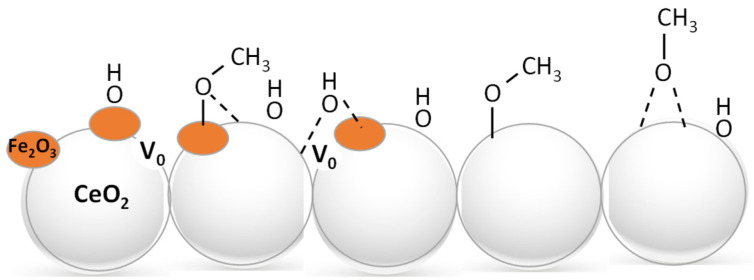
Surface species at the FeCeSH catalyst through methanol adsorption. V_0_: ceria oxygen vacancies.

In sum, our spectroscopic studies on alcohol adsorption indicate that the oxidation activity of the FeCe sample is improved, as suggested by the decrease in the formation of adsorbed partial oxidation products such as carbonyl compounds and CO. These results could support the literature discussion presented in the previous paragraphs, which identifies the doping of ceria with transition metals as a method to increase oxygen vacancies, increasing activity in oxidation processes [22,26]. Although our preparation method based on dry impregnation does not lead to bulk modification, at the surface, the interaction between iron and the ceria support will result in the exposure of new surface sites, such as OH groups, thus adding new adsorption sites able to interact with organic molecules, and a fraction of Fe^3+^ ions, which are themselves able to coordinate and activate organic molecules together with Ce ions (as summarized in Scheme 1). Moreover, doping might also favor the formation of a fraction of Ce^3+^ ions and/or oxygen vacancies due to the interactions occurring at the boundaries between particles in oxide-supported metal oxide catalysts.

Considering the data discussed in the above paragraphs and the previous literature results by Wang et al. [26], a preliminary study has been conducted on the adsorption and activation of 2-chloropropane (2CP), chosen as an example of a chlorinated VOC over the FeCeSH catalyst. At room temperature, the interaction of 2CP with the FeCeSH surface leads to the detection of several IR bands, consistent with molecular adsorption [84]. However, already at 423 K, the band at 1257 cm^−1^ due to C-Cl stretching is disappearing, while new peaks appear, centered at 1160, 1112, 1094, and 930 cm^−1^ (Figure S8), They are assigned to the stretching modes of C-C and C-O bonds and characterize the formation of adsorbed isopropoxide [85]. Their detection points out a fast nucleophilic substitution reaction occurring between the chlorine atoms of the organic substrate and the O^2−^ anion exposed at the surface of the catalyst. This is likely the first activation step for total oxidation of chlorinated compounds, leading to the formation of carbonyl species and a mixture of carboxylate (acetates and formates) and carbonate species adsorbed at the catalyst surface. These preliminary results suggest that on the FeCeHS surface, after the efficient substitution step, chlorinated alkanes are also converted following a common pathway with oxygenated molecules. Indeed, the subsequent evolution of chlorinated species must be carefully evaluated.

### 3.3. Catalytic Activity: Methanol and Ethanol TPSR (MeOH-TPSR and EtOH-TPSR)

In parallel with previous adsorption experiments, and due to the promising IR results, the catalytic behavior of the solids in the oxidation of ethanol and methanol was evaluated by Temperature Programmed Surface Reaction (TPSR) tests. In both cases, oxygen in excess has been used by keeping the C/O_2_ ratio in the range of 1.5–3.0. Tests have been carried out on CeO_2_ (CeHS) and Fe/CeO_2_ (FeCeHS) for both oxygenated feedstocks, i.e., methanol and ethanol.

**Figure 9:**
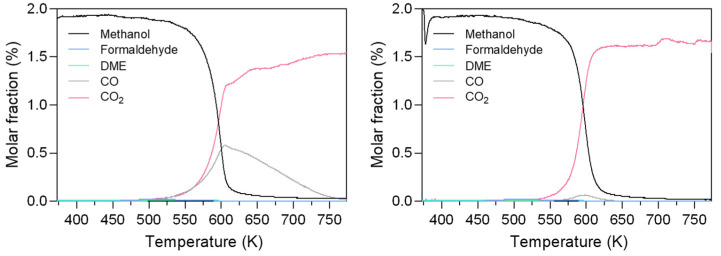
Methanol TPSR results on CeHS (**left**) and FeCeHS (**right**).

In the case of ceria (Figure 9, left), the methanol molar fraction (%) remains constant and approximately equal to 1.9% up to 523 K, where the light-off temperature is set. In fact, above this temperature, it begins to decrease due to the formation of formaldehyde in trace amounts (max. 0.1%, in the temperature range of 440–550 K) and carbon monoxide, whose marked maximum (0.6%) is observed at 603 K. The light-off temperature for CO_2_ is instead observed at 583 K, with a steep increase up to 650 K and then a changing slope up to the maxima observed at the highest tested temperature (1.5%). Methanol conversion reaches mostly 100% above 673 K. For FeCeHS, iron addition changes, in a relevant way, both the catalytic activity and product distribution, resulting in the profiles reported in Figure 9, right. Below 523 K, only small traces of formaldehyde are observed starting from 500 K and are still detectable in the IR spectrum up to 573 K, with a rough maximum molar fraction of 0.2% at 550 K. However, the more relevant component is CO_2_, whose light-off is observed at 523 K, with a mild increase initially in the temperature range up to 590 K that then rapidly achieves the plateau value (1.6%) above 603 K. This suggests that iron introduction does modify the light-off for methanol conversion by producing formaldehyde reasonably by partial oxidation, but it also introduces active sites for the direct full oxidation or fastening CO oxidation, which maximum (0.06%) is observed at 600 K. These findings are likely related to the detection of alkoxy species coordinated over Fe exposed ions, which can affect the reaction path by supporting the oxidation activity of the catalyst even when the surface reduction of ceria starts.

**Figure 10:**
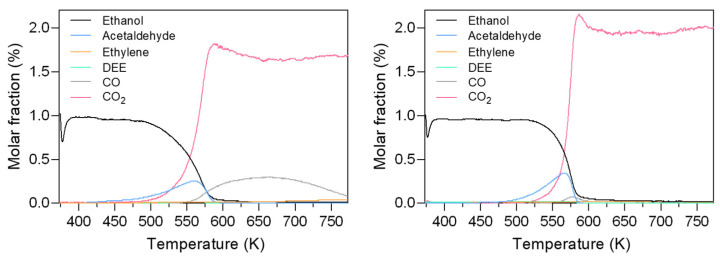
Ethanol TPSR results on CeHS (**left**) and FeCeHS (**right**).

The molar fraction profiles of the reactants and products upon ethanol-TPSR over CeHS and FeCeHS are shown in Figure 10 left and right, respectively. Ethanol did not react below 405 K, where the light-off temperature is observed; in turn, acetaldehyde appeared as a product with a maximum at 553 K and then decreased up to a full disappearance at 593 K.Furthermore, starting from 473 K, where the ethanol profile is quickly reduced, the CO_2_ profile is increased, reaching its maximum value of 1.79% at 583 K and then stabilizing at 1.6% in the whole temperature region. CO formation occurs at temperatures higher than 473 K, reaching a maximum at 663 K (0.3%). Ethylene is observed as a product, in trace amounts, above 673 K but in line with the ceria surface properties discussed above.

In the case of FeCeHS, it can be observed that the light-off is around 473 K where the acetaldehyde molar fraction starts to increase, reaching its maximum at 568 K. Above 520 K, carbon dioxide starts to be observed with a well-defined plateau of 2% reached at around 590 K and is then stabilized at around 1.9%. It is worth mentioning that carbon monoxide is also produced, which again shows a trend with a maximum centered at 578 K, a value considerably lower than that obtained with the CeHS sample and in line with the behavior described in the conversion of C1 alcohol. The formation of methane, ethylene, and diethyl ether (DEE) is not observed.

## 4. Conclusions

High surface ceria and high surface Fe-ceria catalysts were prepared by an easy precipitation–dry–impregnation process and subsequently tested for their surface properties in adsorption-conversion of alcohols. Iron oxide (mainly Fe_2_O_3_) is deposited on the surface of ceria, which maintains its lattice structure, although the particle morphology is slightly changed. Furthermore, the presence of iron oxide increases the heterogeneity of hydroxy groups exposed at the surface.

FT-IR studies show that methanol and ethanol adsorb reactively to the surface of both catalysts, leading to the formation of alkoxides in several coordination states. These species decompose to a mixture of carboxylates and carbonates in the temperature range of 473–673 K. The study of surface species therefore suggests that in the absence of molecular oxygen, both catalysts are active in the combustion process due to the structural lattice oxygen. In the reaction conditions described for the experiments in the IR cell, the formation of carbonyls at the surface is clearly observed in the case of ethanol on CeHS but is significantly limited on FeCeHS.

TPSR tests in the presence of molecular oxygen also confirm that iron addition, even in small amounts, changes both the catalytic activity and product distribution, hindering the formation of formaldehyde from methanol and drastically reducing the quantity of CO produced by the partial oxidation reaction. The FeCeHS catalyst therefore demonstrates, in these particularly severe conditions, good combustion activity in the total oxidation of oxygenated molecules, and a cooperative effect is suggested by the mixture of these two metals in the oxidation process.

## Figures and Tables

**Table 1:** Particle dimensions (Dps) of the powders, and surface area (SA), pore volume (Vp), and pore diameter (dp).

Sample	Dp (μm)			Morphology		
	10%	50%	90%	SA (m^2^ g^−1^)	Vp (cm^3^ g^−1^)	dp (nm)
CeHS	2	9	19	90	0.05	4
FeCeHS	0.3	2	15	53	0.09	9

**Table 2:** Crystallographic and microstructural parameters obtained from the Rietveld method for both samples. Chi-square (χ^2^) values correspond to goodness of fit.

Sample	Phase	Lattice Parameters (Å)		Crystallite Size (nm)	Microstrain (%)	Phase % w	χ^2^ (%)
		a_0_	c_0_				
CeHS	CeO_2_	5.413 (1)		9.1 (1)	0.562 (1)	100	3.59
FeCeHS	CeO_2_	5.409 (1)		9.7 (1)	0.582 (1)	93.0 (3)	3.02
	Fe_2_O_3_	5.040 (1)	13.768 (1)	*	*	7.0 (3)	

* Microstructural parameters were not possible to determine with accuracy due to the low content and crystallinity of the iron oxide; however, they should be comparable to those of CeHS.

## Data Availability

The original contributions presented in this study are included in the article and Supplementary Material. Further inquiries can be directed to the corresponding authors.

## Supplementary Materials

The following supporting information can be downloaded at: https://www.mdpi.com/article/10.3390/ma18040806/s1, Table S1. Structural models used in Rietveld analysis. Table S2. Elemental composition by EDS analysis of the FeCeSH sample. Figure S1. Rietveld refinement of FeCeHS sample in air at room temperature. Observed I (Iobs), calculated I (Icalc), difference between both (Iobs-Icalc), and Bragg positions are indicated. Figure S2. FT-IR skeletal spectra of ceria-based catalysts. Figure S3. Tauc plot of the Kubelka–Munk function (KM) vs. the energy of light absorbed (E) for CeHS (left) and FeCeHS (right) samples. The linear fit of the plot of the fundamental peak is extrapolated to the x-axis. Figure S4. FT-IR subtraction spectra of surface species arising from methanol adsorption over CeHS catalyst. The activated surface spectrum has been subtracted. High frequency region. Figure S5. FT-IR subtraction spectra of surface species arising from methanol adsorption over FeCeHS catalyst. The activated surface spectrum has been subtracted. High frequency region. Figure S6. FT-IR subtraction spectra of surface species arising from ethanol adsorption over CeHS catalyst. The activated surface spectrum has been subtracted. High frequency region. Figure S7. FT-IR subtraction spectra of surface species arising from ethanol adsorption over FeCeHS catalyst. The activated surface spectrum has been subtracted. High frequency region. Figure S8. FT-IR subtraction spectra of surface species arising from 2-Chloropropane adsorption over FeCeHS catalyst after outgassing at room temperature and at increasing temperatures. The activated surface spectrum has been subtracted. CO/CC stretching region.
