# Supplementary material for: A Study of Redox Properties of Ceria and Fe-Ceria Solid Materials Through Small Molecules Catalytic Oxidation
Source: Materials (Basel). 2025 Feb 12;18(4):806. doi: 10.3390/ma18040806 (PMC11857746; doi:10.3390/ma18040806)
Supplement: Supplementary file 1 [file materials-18-00806-s001.zip › materials-3391636-supplementary.pdf]

Article

A Study of Redox Properties of Ceria and Fe-Ceria Solid Materials Through Small Molecules Catalytic Oxidation

Riccardo Balzarotti [1], Andrea Basso Peressut [2], Gabriella Garbarino [3], Elena Spennati [3], Juan Felipe Basbus [3], Maria Paola Carpanese [3], Saverio Latorrata [2], Cinzia Cristiani [2,]* and Elisabetta Finocchio [3,]*

[1] Department of Innovative Technologies, University of Applied Sciences and Arts of Southern Switzerland, Via la Santa 1, 6962 Lugano, Switzerland; riccardo.balzarotti@supsi.ch

[2] Department of Chemistry, Materials and Chemical Engineering "G. Natta", Politecnico di Milano, Piazza Leonardo da Vinci 32, 20133 Milan, Italy; andreastefano.basso@polimi.it (A.B.P.); saverio.latorrata@polimi.it (S.L.)

[3] Department of Civil, Chemical and Environmental Engineering (DICCA), University of Genova (UniGe), Via All'opera Pia 15, 16145 Genoa, Italy; gabriella.garbarino@unige.it (G.G.); elena.spennati@edu.unige.it (E.S.); juanfelipe.basbus@edu.unige.it (J.F.B.); maria.paola.carpanese@unige.it (M.P.C.)

* Correspondence: cinzia.cristiani@polimi.it (C.C.); elisabetta.finocchio@unige.it (E.F.)

Supporting Info.

Table S1. Structural models used in Rietveld analysis.

Phase	Atom	Space Group	Wyckoff positions	Occupancy	Atomic positions		
					x	y	z
CeO_2	Ce	Fm-3m	4a	1	0	0	0
	O		8c	1	0.25	0.25	0.25
Fe_2O_3	Fe	R-3c	12c	1	0	0	0.355
	O		18e	1	0.694	0	0.25

Table S2. Elemental composition by EDS analysis of the FeCeSH sample.

Element	Atomic concentration (%)	Oxide	Weight percentage (%)
Ce	38.63	CeO_2	90.72
Fe	6.01	Fe_2O_3	6.95
O	38.89	-	–
N	1.13	-	–
C	15.34	-	–

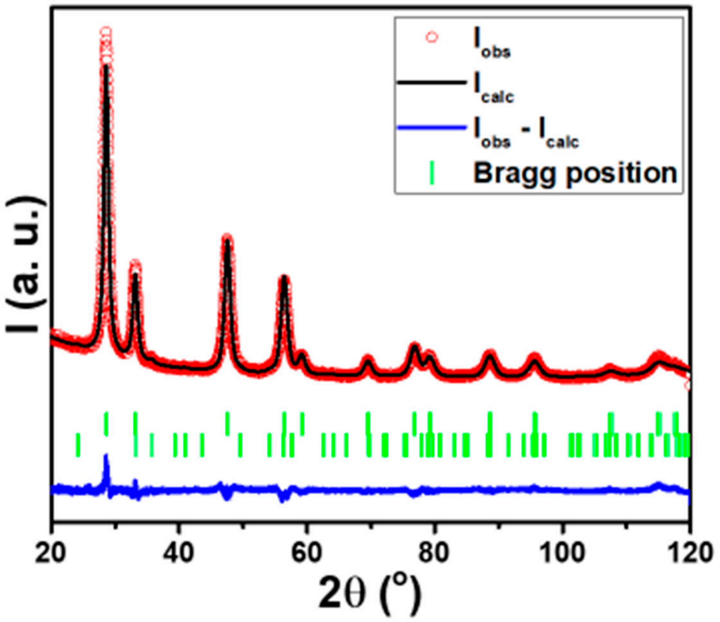

Figure S1. Rietveld refinement for FeCeHS sample in air at room temperature. Observed I (I_{obs}), calculated I (I_{calc}), difference between both (I_{obs} - I_{calc}), and Bragg positions are indicated.

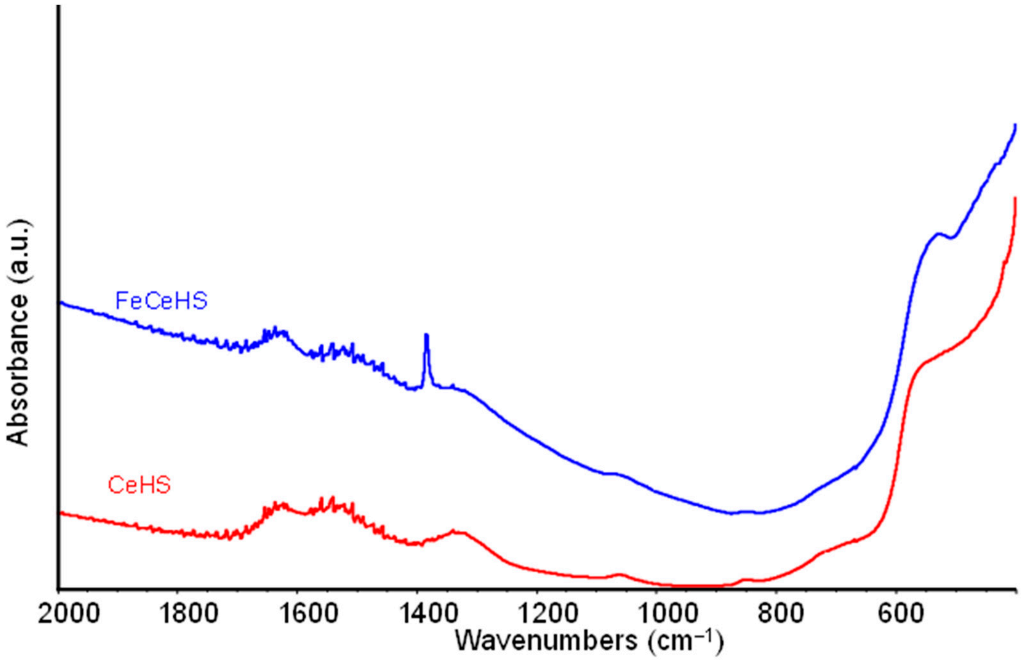

Figure S2. FT IR skeletal spectra of ceria-based catalysts.

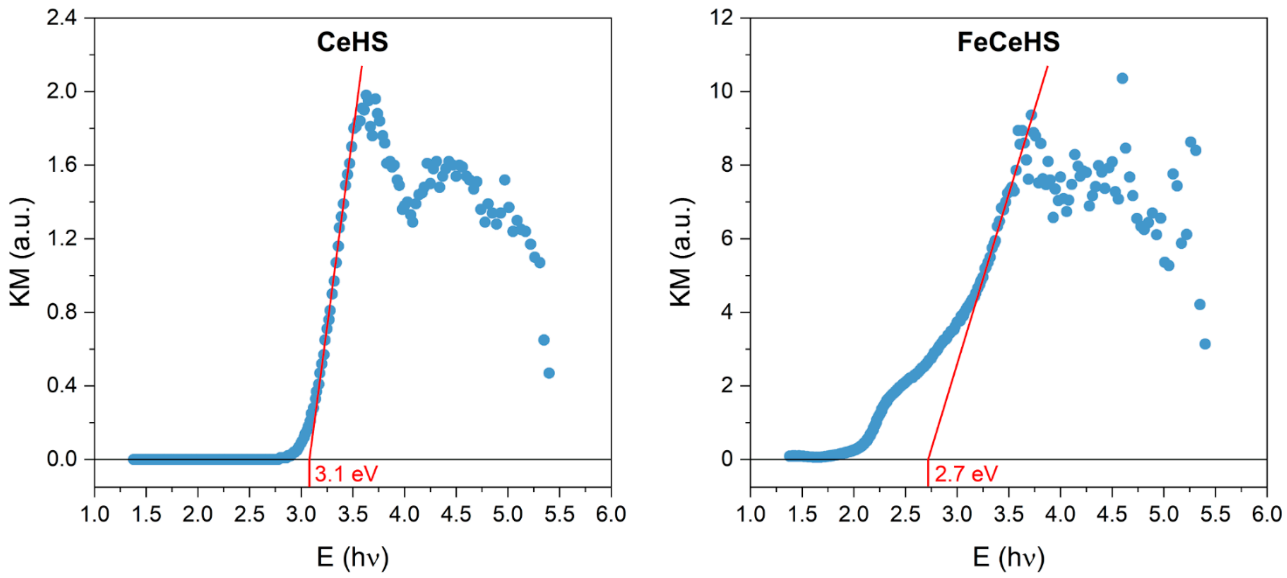

Figure S3. Tauc plot of the Kubelka-Munk function (KM) vs. the Energy of light absorbed (E) for CeHS (left) and FeCeHS (right) samples. The linear fit of the plot of the fundamental peak is extrapolated to the x-axis.

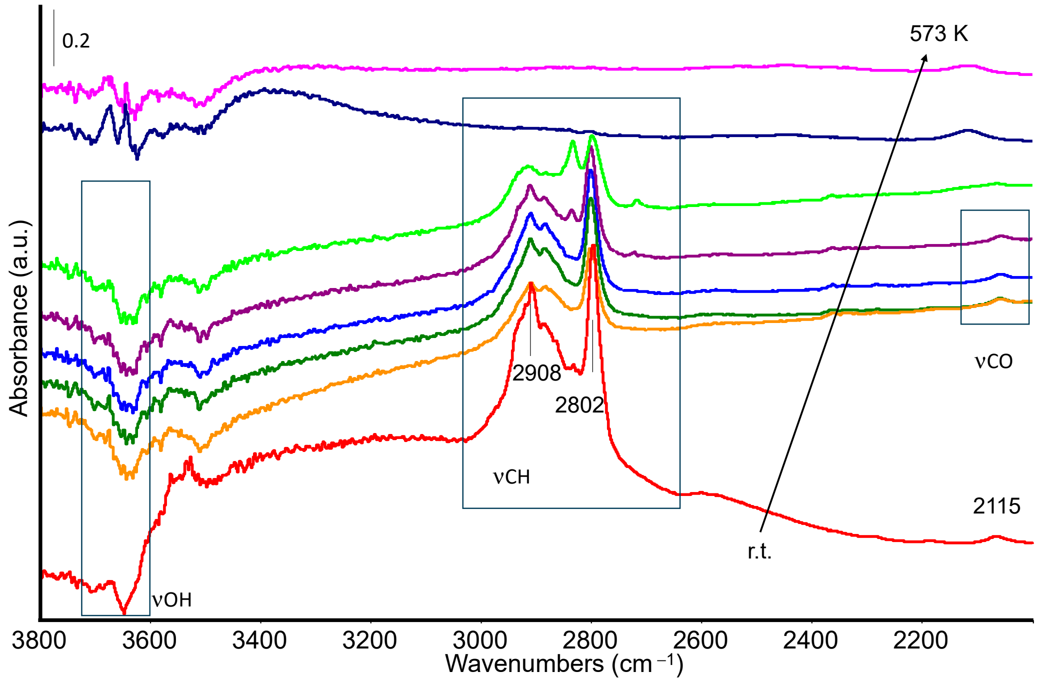

Figure S4. FT IR subtraction spectra of surface species arising from methanol adsorption over CeHS catalyst. The activated surface spectrum has been subtracted. High frequency region.

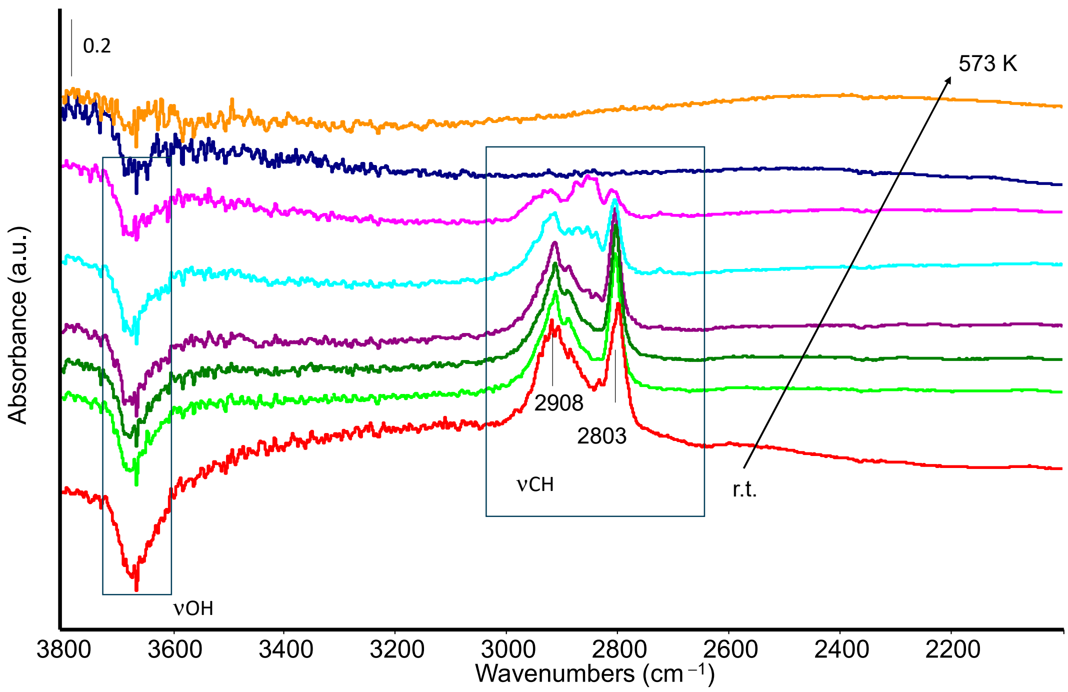

Figure S5. FT IR subtraction spectra of surface species arising from methanol adsorption over FeCeHS catalyst. The activated surface spectrum has been subtracted. High frequency region.

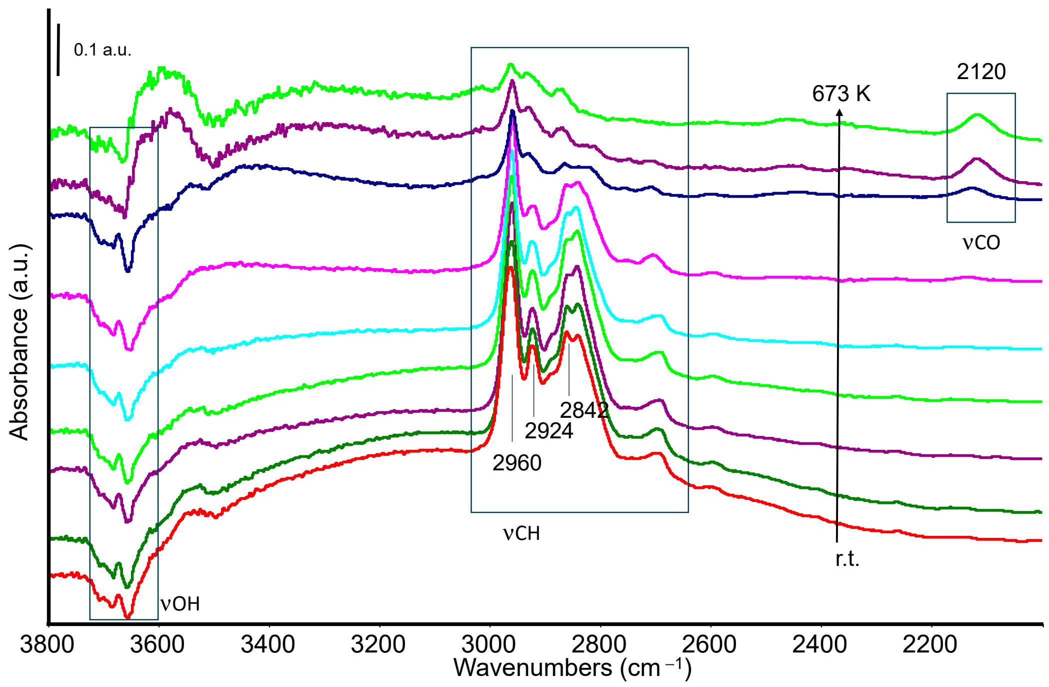

Figure S6. FT IR subtraction spectra of surface species arising from ethanol adsorption over CeHS catalyst. The activated surface spectrum has been subtracted. High frequency region.

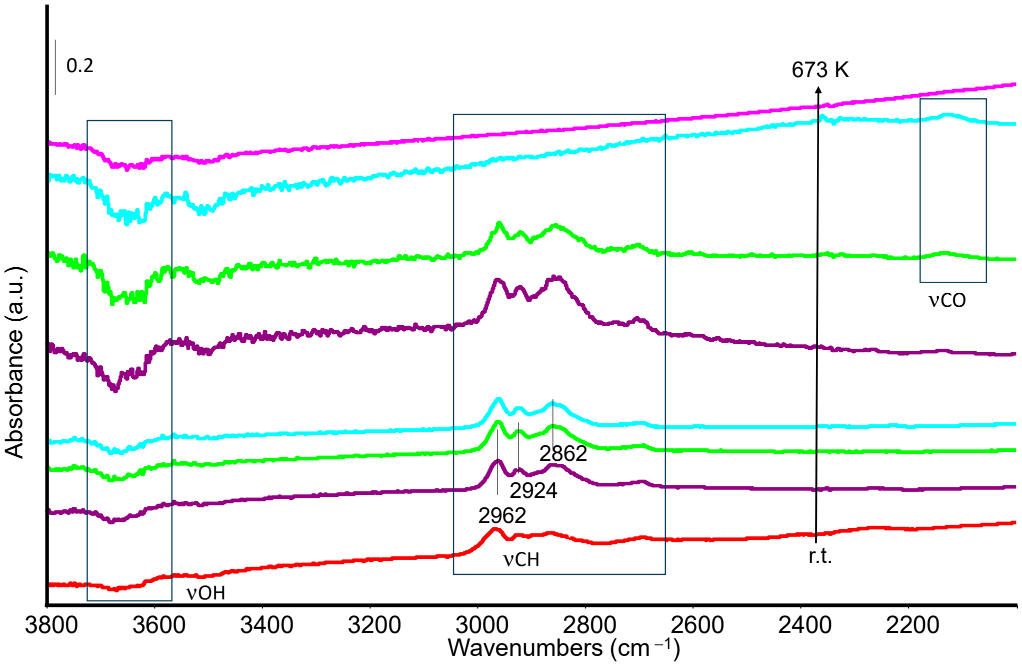

Figure S7. FT IR subtraction spectra of surface species arising from ethanol adsorption over FeCeHS catalyst. The activated surface spectrum has been subtracted. High frequency region.

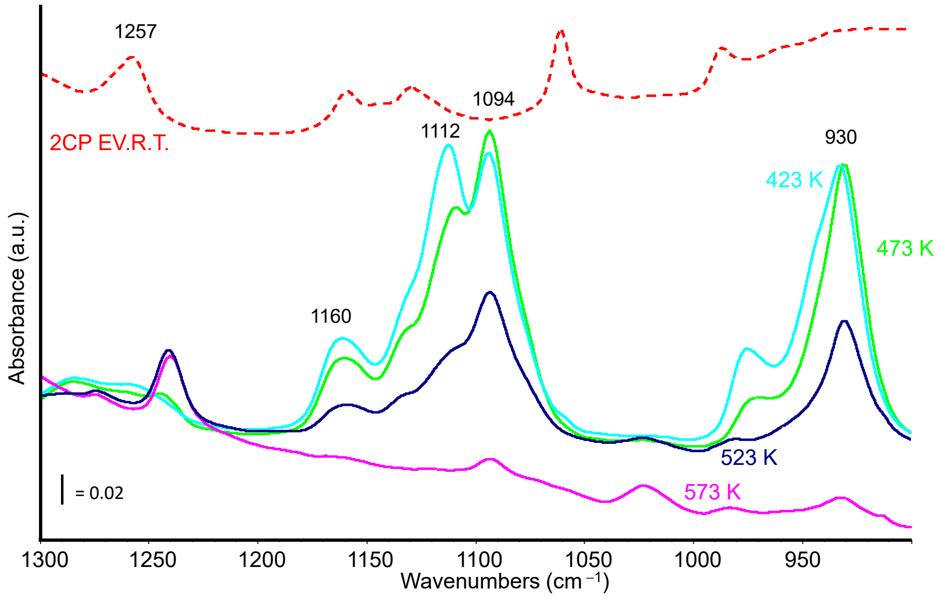

Figure S8. FT IR subtraction spectra of surface species arising from 2-Chloropropane adsorption over FeCeHS catalyst after outgassing at room temperature and at increasing temperatures. The activated surface spectrum has been subtracted. CO/CC stretching region.
